# Internet of Things for Smart Community Solutions

**DOI:** 10.3390/s22020640

**Published:** 2022-01-14

**Authors:** Dhananjay Singh, Mario Divan, Madhusudan Singh

**Affiliations:** 1 Department of Electronics Engineering, Hankuk University of Foreign Studies (HUFS), Yongin 17035, Korea; dsingh@hufs.ac.kr; 2 Department of Information System, National Technological University, Córdoba 1826, Argentina; mjdivan@eco.unlpam.edu.ar; 3 Endicott College of International Studies, Woosong University, Daejeon 34606, Korea

The term IoT (Internet of Things) constitutes the quickly developing advanced gadgets with highest computing power with in a constrained VLSI design space. It is called things on the internet because, this whole ecosystem of device communication and exchange of information is hosted on internet (interconnected networks using standardized communication protocols). The IoT assimilate smart sensors and gadgets on the web infrastructure worldwide. This collaboration of smart gadgets and web worldwide allows to remotely control the system of IoT. IoT offers multi-operation degree for urban areas to utilize information to oversee traffic, cut contamination, utilize foundation and keep residents protected and clean. 

## 1. Summary of the Special Issue

The works submitted to this special issue to enhance the IoT infrastructure and develop more dynamic system, following works has been noted as the most significant in this domain.

In [1], author proposed an offline feature extraction model to process and analyze log data acquired from IoT and wireless devices to improve and enhance the security systems, performance and manage IoT system. The proposed model LogEvent2vec gathers log events as an input and vectorize the log event. After getting the log event vector, the log event vector is transformed into the log sequence vector by bary or tf-idf. This novel offline feature extraction approach word2vec extracts the relevance between log events, and reduce the time of training and coordinate transformation. The designed framework model is trained using supervised models (Random Forests, Naive Bayes, and Neural Networks) to perform anomaly detection.

The general framework of log anomaly detection consists of three steps: log parsing, feature extraction, and anomaly detection. The log parsing is used to remove all specific parameters from log messages and extract all the log events. Log events consists of text and numerical data, where textual data requires to be numerically encoded for successful anomaly detection. NLP model is deployed to encode data numerically along with word2vec for feature extraction. The feature vectors of log sequence are trained with machine learning and deep learning models for anomaly detection. Then, the trained model predicts whether a new log sequence is anomalous or not. The main advantage of LogEvent2vec architecture is that it can work with any coordinate transformation methods and anomaly detection models. The experiment conducted on real public log dataset has achieved numerous milestones in the likes of reduced computation time and higher accuracy.

The work developed in [2] shows an energy efficient method for clustering and routing in WSNs (wireless sensor networks) to increase the lifetime of the wireless networks in the case of disaster management in smart cities. In major disaster events like forest fire, weather situation are non-uniform and has uncontrolled behavior which is why they spread very quickly. For these unprecedented events, authors have proposed an improved hybrid PSO–HSA-based CH selection method with a novel fitness function including energy-efficiency, cluster closeness, and network coverage criteria. The proposed method allows to select relay nodes in WSN based on multi-hop routing with a fitness function including energy-efficiency and communication link quality criteria. On successful mapping of nodes, AWS mathematical model is used to solve the fitness function to lower the cost of optimization.

The main objective of this work was to design an energy efficient clustering and routing method for IoT network devices. They employed PSO to select relay nodes with a fitness function which also resulted in the development of a modified tree encoding method based on a new data packet format. The new data packet format is adapted to be used in disaster applications; furthermore, modifying the PSO encoding priority-based routing to build an optimal routing tree with non-uniform events. The entire architecture of data packet transformation from sensor nodes to base station, their energy consumption, active sensor nodes in the disaster events like forest fire can be monitored and improved with presented methodology.

In [3], authors discussed the challenges in vehicle delay tolerant networks (VDTN) and review individual nodes and their incentive approach to make data transfer successful with all the constrains being onsite. To establish end-to-end successful communication, nodes needs to forward message to the destination but all the nodes in the network do not cooperate and contribute their resources for message forwarding without any reward. This is where the term coined as selfish nodes in the network architecture comes in. These selfish nods restrain to utilize their resources and do not cooperate to forward the messages, and are inclined to increase their own resources. This is described as the major challenge in VDTNs and author has presented this paper to study and examine various incentives approach deployed on the network for these selfish nodes to forward message. The selfish behavior of nodes are classified into two different types, namely, collusion and non-cooperation. Also the incentive schemes are classified into four different types, namely, credit-based, reputation-based, barter-based system, and game-theoretic incentive schemes. These incentive schemes encourage selfish nodes to participate in the network activities and reward them on every transaction (message forward).

The primary aim of this paper is to review different papers in the field of delay tolerant networks technology and analyze and classify them according to the type of incentive techniques used to encourage node for cooperation within a network. The documents studied to analyze incentive techniques are compared thoroughly and summarized on the parameters of cost, approach, detected nodes, scalability, detection accuracy, buffer consumption, and applicability.

In [4], authors proposed a methodology for device-to-device D2D communication in a hybrid infrastructure where On-Scene Available (OSA) user equipment’s on the network can be connected seamlessly to establish end-to end multi-hop communication between and command center. Challenges in the end-to-end network connectivity is addressed by optimization of Energy and Spectral efficiency (ESE) in a scenario in which multi-hop D2D connections can provide diffused connectivity without a centralized support, keeping a low complexity and given the respect of constraints regarding the power, number of hops and mutual interference. They proposed a dynamic adaptation approach based on machine learning to improve a joint energy-spectral efficiency (ESE). In this proposed framework, Q-learning enhance ESE measure, also learner agents & scheduler agents by applying it in a hybrid fashion. To apply Q-learning on learner agents and scheduler agents, next hop selection and RRH selection algorithms are proposed.

The proposed ML-based approach finds out the best path by considering the network parameters, i.e., RRH load, congestion level, link quality metric, average number of hops, and throughput. The routing path is optimized to make the learner agents work and behave efficiently so that both energy and spectral efficiency are enhanced simultaneously and dynamically. Authors have compared their approach results with baseline algorithm and outperformed former by 67% in terms of joint ESE. The results show also that proposed framework with C-RAN reduces latency by approximately 50% w.r.t. the baseline.

In [5] authors presented a design of distributed group location update algorithm (DGLU) which alleviates concurrent problems such as signaling overhead in networks and energy depletion of IoT devices in massive machine type communication (mMTC) systems. This proposed system can geographically proximate IoT devices movement to other location and conducts location update in a distributed manner. To enact this novel technique of group location update, authors have formulated a constrained stochastic game model that can be converted into an equivalent linear programming (LP). They have also designed a best response dynamics-based algorithm to reach a multi-policy constrained Nash equilibrium. The work done in this paper is one of its first work to optimize the trade-off between the accuracy of location information and the energy efficiency of IoT devices in a distributed manner. This complete framework is based on a constrained stochastic game model which helps to achieve optimal location update with few iterations.

The results after evaluation signifies that DGLU can achieve an accuracy of location information that is comparable with that of the individual location update scheme while guaranteeing a low energy outage probability. Results also demonstrates that proposed algorithm is not high, and DGLU operates adaptively even when the operating environments change.

## 2. IoT Application and Methodologies for Improvement

Internet of things solutions has steadily penetrated into a myriad of fields. In this special issue, many works have been submitted with regard to the application of IoT web infrastructure and improvements in those respective approach. Works related to health care and their realtime monitoring to slove real life difficulties has been issues in the same. Novel approach combining smart IoT and blockchain as well as methodologies to prevent possible attacks methodologies has been discussed in the works submitted in this special issue.

In [6], author designed a model to monitor the working of medical equipment to detect disease and manage patient health remotely. This work especially targets specific healthcare domain i.e. kidney for their treatment and to establish transparent and trustworthy healthcare system. Initially, authors designed a model for normal kidney to examine their operation, its parts, and possibilities of disease. Thereafter, a model is designed and analyzed for a wearable kidney system. A wearable kidney system mirrors lively kidney in a designed framework and gathers records of kidney functionalities in the form of transaction. The blockchain system is integrated in the framework model to obtain data of various parts (or in this wearable kidney different components) in the form of transaction and constructs blockchain. Then the data is transferred to remote location for analysis and subsequent medication through Industry 4 processes. The industry 4 process involves data analysis, interpretation, secure exchange, and provide timely information to patients and doctors as well.

The main objective of this designed healthcare model is to integrate Healthcare 4.0 processes with a patient-centric healthcare system. Healthcare 4.0 includes Industry 4.0 processes (Internet of Things (IoT), Industrial IoT (IIoT), cognitive computing, machine learning, AI, etc.) integrated with the healthcare system. To integrate consensus algorithm in the blockchain network of the proposed model, a blockchain-based consensus algorithm with game theory is proposed for healthcare. The study and experiments conducted behind integration of normal kidney, wearable kidney, healthcare 4.0 and blockhain has numerous advantage in electronic medical records, supply systems, biomedical research, and education, data analytics etc.

The work in [7] proposes a novel approach for continuous monitoring of driver’s activities, motion and subsequently analysis of road infrastructure to prevent unfortunate events such as accidents and also alerts during drowsiness or distraction while driving. The state-of-the-art behavior prediction system using deep learning model is introduced, namely dynamic driver profile (DDP) to identify drivers risk pattern. This system analyzes the current state of driver, interpolates past behavioral data and with the help of recommendation based on deep learning methods, it enables automatic interventions for the safety of the driver. The proposed SafeDrive architecture hosts several behavioral sensors for the driver’s driving prediction and the data collected from these sensors is transferred to the centralized cloud server. The process of data transfer is completed in three where first tire includes intelligent connected vehicles along inbuilt sensors, OBU, and E-RSUs. Seconds tier includes distributed fog nodes which serves the responsibility of monitoring and controlling of each components in tier 1. And the third is the uppermost tier which includes centralized cloud server facility which houses the continuous monitoring data and informatics obtained from tier 1 and tier 2 devices. This proposed architecture is also called as hybrid recommendation system architecture which aims to prevent accidents and generate alert alarm in the connected vehicle environment. 

Authors also presented a deep learning model to train the prediction model with the driver’s prehistoric data of accident and violent records from DDP. On the successive contrary, data classification can be achieved through proposed deep learning design and it can also be updated on cloud server for successive epochs for continuous monitoring in future. Some preliminary scenarios and experiment results are also presented which helps to improve the driving behavior.

The work in [8] investigates the security of the general model of PoS (Proof of Stake) protocol in the blockchain environment against double-spend attack. Double spend attack is realized on blockchain environment when coins are transferred by attackers to their own accounts during transaction for goods and services, at the same time an alternative chain is created longer enough to guarantee acceptance among honest miners. During the block generation, these attackers hide their alternative branch of block until the necessary number of confirmation blocks are created, since they themselves create the chain of blocks, during selfish mining, while honest miners see the alternative chain and occasionally may maintain its creation. 

The work done in this paper assumes condition which is the probability that the next block is generated by some stakeholder is proportional to its stake. The success of transaction is confirmed when the probability of its success is obtained, which depends on the network parameters and the number of confirmation blocks. For the successful confirmation of block, authors gave two strategies in form of mathematical expression and concluded strategy 1 as most preferable again adversaries than strategy 2.

In [9], authors proposed a hidden Markov model (HMM) based framework to pre-empt the exigent state of an affliction and lower the chances of complications and mortality through intelligence-backed decisions. The proposed framework model was trained over the symptoms data of SARS-Cov-2 from Welltory repository and reading of body vitals using Apple Watch, Fitbit, and Garmin smart bands. Hidden Markov Model (HMM) machine learning-based framework was designed to predict the onset and increased severity of an infection such as SARS-Cov-2. HRV (Herat Rate Variability) measurements was also used by an algorithm due to its applicability in pre-emptive diagnostics. The hidden states of the HMM comprise changes in the standard deviation of NN (SSDN), rMSSD, and the low frequency (LF), high frequency (HF), and very low frequency (VLF) components of the HRV measurements that the participants had recorded during the course of the study.

The main principle to design this framework model was for monitoring the trends followed by the use of HRV time and frequency based metrics to predict the onset and oncoming exigent state of a major illness. The framework architecture has two main components, data processing and model training which uses HRV components to discern the hidden states. The Viterbi algorithm was used to generate sequences of transitions between the hidden states, and the outputs were found to agree with the transitions detected from the HRV readings. The result from HMM model suggests that a consistent decline in the values of major HRV components could be attributed to the onset or worsening of SARS-Cov-2 infection.

In [10], various works related to civil engineering and construction sector is studied and the role of artificial intelligence (AI) in the same field is highlighted. In this review analysis, works from 2005 to 2020 has been studied concerning various applications of AI in civil engineering, especially focused on construction. Authors conducted the literature review of various articles, research papers and works in civil engineering related to AI techniques for the prediction of soil shear strength and pre-project cost and duration. From the survey conducted, authors found that, among the various AI models and techniques, ANN was the most used model followed by regression and SVM. ANN’s ability to provide a generalized optimal solution with high accuracy in less time and its ease of implementation on various platform, boosted its use universally. The performance evaluation of various AI techniques in the prediction of soil shear strength and pre-project cost and duration, metrics such as RMSE, correlation coefficient (R), MAPE, MAE, coefficient of determination (R2), VAF, and AAE were the most used metrics.

Literature review recommends that accurate estimation of soil shear strength, input parameters such as the percentage of clay, plastic index, liquid limit, sand percentage, plastic limit, wet density of the soil, silt percentage, and dry density are crucial. Project cost is the most volatile and is greatly affected due to varying input parameters such as project duration, project type (bridge, highway, or others), project size, location, geographical complexity, road length and width, soil condition, etc. Internal as well as external factors (also listed in paper) can lead to cost overrun. After detailed analysis of several research papers, articles and works, authors suggests to design an AI model in a robust and adaptive manner to work efficiently even if some of the input parameters are missing. The aim of this review article was to assist researchers and civil engineers in determining the strength and potential of AI in the construction sector, which will eventually assist them in automating the manual, repetitive, and time-consuming tasks with high precision and lower effort and cost.

## Data Availability

Not applicable.
